# Ethyl 1-phenyl-2-[4-(trifluoro­meth­oxy)phen­yl]-1*H*-benzimidazole-5-carboxyl­ate

**DOI:** 10.1107/S1600536812034903

**Published:** 2012-08-15

**Authors:** Yeong Keng Yoon, Mohamed Ashraf Ali, Tan Soo Choon, Suhana Arshad, Ibrahim Abdul Razak

**Affiliations:** a Institute for Research in Molecular Medicine, Universiti Sains Malaysia, Minden 11800, Penang, Malaysia; b School of Physics, Universiti Sains Malaysia, 11800 USM, Penang, Malaysia

## Abstract

In the title compound, C_23_H_17_F_3_N_2_O_3_, an intra­molecular C—H⋯F hydrogen bond generates an *S*(6) ring motif. The essentially planar 1*H*-benzimidazole ring system [maximum deviation = 0.021 (2) Å] forms dihedral angles of 25.00 (10) and 62.53 (11)° with the trifluoro­meth­oxy-substituted benzene and phenyl rings, respectively. The twist of the ethyl acetate group from the least-squares plane of the 1*H*-benzimidazole ring system is defined by a C(=O)—O—C—C torsion angle of 79.5 (3)°. In the crystal, mol­ecules are linked into a two-dimensional network parallel to the *bc* plane by weak C—H⋯N and C—H⋯O hydrogen bonds. Weak C—H⋯π inter­actions also observed.

## Related literature

For the biological activity of benzimidazoles, see: Lemura *et al.* (1986 ▶); Zhang *et al.* (2008 ▶). For related structures, see: Yoon *et al.* (2011 ▶, 2012*a* ▶,*b* ▶,*c* ▶). For hydrogen-bond motifs, see: Bernstein *et al.* (1995 ▶). For the stability of the temperature controller used in the data collection, see: Cosier & Glazer (1986 ▶).
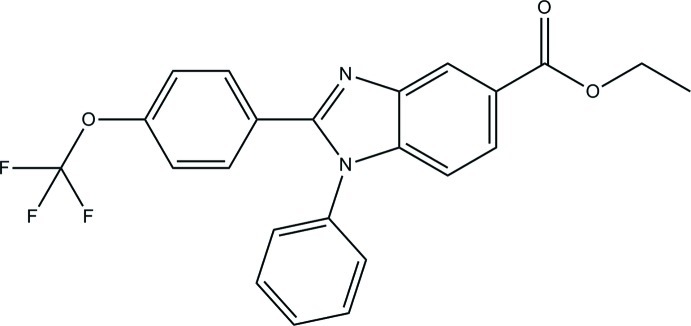


## Experimental

### 

#### Crystal data

C_23_H_17_F_3_N_2_O_3_
*M* *_r_* = 426.39
Triclinic, 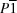

*a* = 8.0204 (4) Å
*b* = 10.8943 (6) Å
*c* = 11.4329 (7) Å
α = 76.706 (4)°
β = 83.269 (4)°
γ = 81.227 (4)°
*V* = 957.31 (9) Å^3^
*Z* = 2
Mo *K*α radiation
μ = 0.12 mm^−1^
*T* = 100 K
0.37 × 0.27 × 0.20 mm

#### Data collection

Bruker SMART APEXII CCD area-detector diffractometer
Absorption correction: multi-scan (*SADABS*; Bruker, 2009 ▶) *T*_min_ = 0.957, *T*_max_ = 0.977
12437 measured reflections
4388 independent reflections
3028 reflections with *I* > 2σ(*I*)
*R*_int_ = 0.065

#### Refinement

*R*[*F* ^2^ > 2σ(*F* ^2^)] = 0.075
*wR*(*F* ^2^) = 0.206
*S* = 1.05
4388 reflections
281 parameters
H-atom parameters constrained
Δρ_max_ = 0.87 e Å^−3^
Δρ_min_ = −0.42 e Å^−3^

### 

Data collection: *APEX2* (Bruker, 2009 ▶); cell refinement: *SAINT* (Bruker, 2009 ▶); data reduction: *SAINT*; program(s) used to solve structure: *SHELXTL* (Sheldrick, 2008 ▶); program(s) used to refine structure: *SHELXTL*; molecular graphics: *SHELXTL*; software used to prepare material for publication: *SHELXTL* and *PLATON* (Spek, 2009 ▶).

## Supplementary Material

Crystal structure: contains datablock(s) global, I. DOI: 10.1107/S1600536812034903/lh5511sup1.cif

Structure factors: contains datablock(s) I. DOI: 10.1107/S1600536812034903/lh5511Isup2.hkl

Supplementary material file. DOI: 10.1107/S1600536812034903/lh5511Isup3.cml

Additional supplementary materials:  crystallographic information; 3D view; checkCIF report

## Figures and Tables

**Table 1:** Hydrogen-bond geometry (Å, °) *Cg*1 and *Cg*2 are the centroids of the C14–C19 and N1/N2/C1/C6/C7 rings, respectively.

*D*—H⋯*A*	*D*—H	H⋯*A*	*D*⋯*A*	*D*—H⋯*A*
C12—H12*A*⋯F2	0.95	2.37	2.956 (3)	120
C15—H15*A*⋯N1^i^	0.95	2.56	3.490 (3)	167
C18—H18*A*⋯O2^ii^	0.95	2.40	3.307 (4)	160
C19—H19*A*⋯O2^iii^	0.95	2.50	3.412 (3)	160
C13—H13*A*⋯*Cg*1	0.95	2.79	3.592 (3)	142
C21—H21*A*⋯*Cg*2^iii^	0.99	2.95	3.634 (3)	127

Symmetry codes: (i) 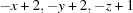; (ii) 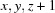; (iii) 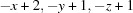.

**Fig. 1.**
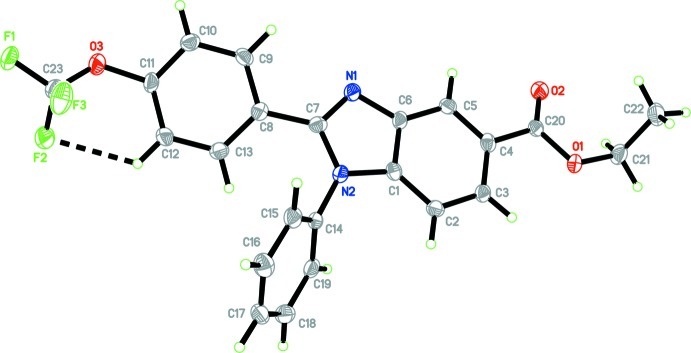
The molecular structure of the title compound, showing 50% probability displacement ellipsoids. The dashed line indicates a weak hydrogen bond.

**Fig. 2.**
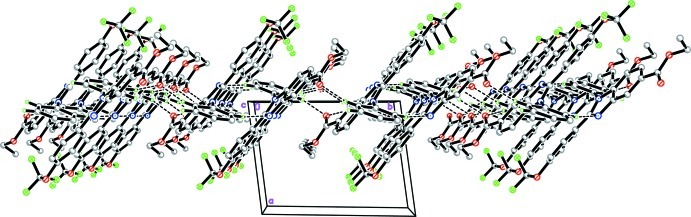
The crystal packing of the title compound viewed along the c-axis. The H atoms not involved in the intermolecular interactions (dashed lines) have been omitted for clarity.

## supplementary crystallographic information

### Comment

Benzimidazole-based heterocycles are of wide interest because of their diverse biological activities and various clinical applications. Among various applications, they are known to exhibit antihistamine (Lemura *et al.*, 1986) and immunosuppressive (Zhang *et al.*, 2008) activities. As part of our ongoing structural studies of benzimidazole derivatives (Yoon *et al.*, 2011), we report the structure of the title compound.

The molecular structure is shown in Fig. 1. Bond lengths and angles are within normal ranges and are comparable to related structures (Yoon *et al.*, 2012*a*,*b*,*c*). An intramolecular C12—H12A···F2 hydrogen bond generates an *S*(6) ring motif (Bernstein *et al.*, 1995). The essentially planar 1*H*-benzimidazole ring system [N1/N2/C1–C7, with a maximum deviation of 0.021 (2) Å at atoms C6 and C7] is inclined to the trifluoromethoxy-substituted benzene ring (C8–C13) and the pendant phenyl ring (C14–C19), making dihedral angles of 25.00 (10) and 62.53 (11)°, respectively. The ethyl acetate group (O1/O2/C20–C22) is twisted away from the least-square plane of the 1*H*-benzimidazole ring at the O1–C21 bond, as indicated by the torsion angle C20—O1—C21—C22 = 79.5 (3)°.

The crystal packing is shown in Fig. 2. Weak intermolecular C15—H15A···N1^i^, C18—H18A···O2^ii^ and C19—H19A···O2^iii^ (Table 1) hydrogen bonds link the molecules into a two-dimensional network parallel to the *bc*-plane. The crystal structure is further stabilized by weak intermolecular C13—H13A···*Cg*1 and C21—H21A···*Cg*2 (Table 1) interactions (*Cg*1 and *Cg*2 are the centroids of C14–C19 and N1/N2/C1/C6/C7 rings, respectively).

### Experimental

Ethyl 3-amino-4-(phenyl amino) benzoate (0.84 mmol) and the sodium metabisulfite adduct of trifluoromethoxy benzaldehyde (1.68 mmol) were dissolved in DMF. The reaction mixture was refluxed at 403K for 2 h. After completion, the reaction mixture was diluted in ethyl acetate (20 ml) and washed with water (20 ml). The organic layer was collected, dried over Na_2_SO_4_ and the evaporated *in vacuo* to yield the product. Crystals were obtained from a solution of the title compound in ethyl acetate.

### Refinement

All H atoms were positioned geometrically [C–H = 0.95–0.99 Å] and refined using a riding model with *U*_iso_(H) = 1.2 *U*_eq_(C) or *U*_iso_(H) = 1.5 *U*_eq_(C_methyl_). A rotating group model was applied to the methyl group hydrogen atoms.

### Crystal data

**Table:**

C_23_H_17_F_3_N_2_O_3_	*Z* = 2
*M**_r_* = 426.39	*F*(000) = 440
Triclinic, *P*1	*D*_x_ = 1.479 Mg m^−^^3^
Hall symbol: -P 1	Mo *K*α radiation, λ = 0.71073 Å
*a* = 8.0204 (4) Å	Cell parameters from 2463 reflections
*b* = 10.8943 (6) Å	θ = 2.6–29.7°
*c* = 11.4329 (7) Å	µ = 0.12 mm^−^^1^
α = 76.706 (4)°	*T* = 100 K
β = 83.269 (4)°	Block, colourless
γ = 81.227 (4)°	0.37 × 0.27 × 0.20 mm
*V* = 957.31 (9) Å^3^	

### Data collection

**Table:**

Bruker SMART APEXII CCD area-detector diffractometer	4388 independent reflections
Radiation source: fine-focus sealed tube	3028 reflections with *I* > 2σ(*I*)
Graphite monochromator	*R*_int_ = 0.065
φ and ω scans	θ_max_ = 27.5°, θ_min_ = 1.8°
Absorption correction: multi-scan (*SADABS*; Bruker, 2009)	*h* = −10→10
*T*_min_ = 0.957, *T*_max_ = 0.977	*k* = −14→14
12437 measured reflections	*l* = −14→12

### Refinement

**Table:**

Refinement on *F*^2^	Primary atom site location: structure-invariant direct methods
Least-squares matrix: full	Secondary atom site location: difference Fourier map
*R*[*F*^2^ > 2σ(*F*^2^)] = 0.075	Hydrogen site location: inferred from neighbouring sites
*wR*(*F*^2^) = 0.206	H-atom parameters constrained
*S* = 1.05	*w* = 1/[σ^2^(*F*_o_^2^) + (0.123*P*)^2^] where *P* = (*F*_o_^2^ + 2*F*_c_^2^)/3
4388 reflections	(Δ/σ)_max_ < 0.001
281 parameters	Δρ_max_ = 0.87 e Å^−^^3^
0 restraints	Δρ_min_ = −0.42 e Å^−^^3^

### Special details

**Table:**

Experimental. The crystal was placed in the cold stream of an Oxford Cryosystems Cobra open-flow nitrogen cryostat (Cosier & Glazer, 1986) operating at 100.0 (1) K.
Geometry. All e.s.d.'s (except the e.s.d. in the dihedral angle between two l.s. planes) are estimated using the full covariance matrix. The cell e.s.d.'s are taken into account individually in the estimation of e.s.d.'s in distances, angles and torsion angles; correlations between e.s.d.'s in cell parameters are only used when they are defined by crystal symmetry. An approximate (isotropic) treatment of cell e.s.d.'s is used for estimating e.s.d.'s involving l.s. planes.
Refinement. Refinement of *F*^2^ against ALL reflections. The weighted *R*-factor *wR* and goodness of fit *S* are based on *F*^2^, conventional *R*-factors *R* are based on *F*, with *F* set to zero for negative *F*^2^. The threshold expression of *F*^2^ > σ(*F*^2^) is used only for calculating *R*-factors(gt) *etc*. and is not relevant to the choice of reflections for refinement. *R*-factors based on *F*^2^ are statistically about twice as large as those based on *F*, and *R*- factors based on ALL data will be even larger.

### Fractional atomic coordinates and isotropic or equivalent isotropic displacement parameters (Å^2^)

**Table:**

	*x*	*y*	*z*	*U*_iso_*/*U*_eq_	
F1	0.2469 (2)	1.38071 (15)	0.84048 (15)	0.0330 (4)	
F2	0.3445 (2)	1.20231 (16)	0.94936 (15)	0.0353 (5)	
F3	0.5165 (2)	1.32405 (16)	0.84269 (18)	0.0379 (5)	
O1	1.3555 (2)	0.41546 (17)	0.33931 (16)	0.0229 (4)	
O2	1.1298 (2)	0.50753 (18)	0.23637 (17)	0.0247 (4)	
O3	0.3587 (2)	1.23201 (19)	0.74959 (18)	0.0296 (5)	
N1	0.8708 (3)	0.8508 (2)	0.4871 (2)	0.0207 (5)	
N2	1.0123 (3)	0.8059 (2)	0.65459 (19)	0.0194 (5)	
C1	1.0871 (3)	0.7205 (2)	0.5846 (2)	0.0185 (5)	
C2	1.2206 (3)	0.6226 (2)	0.6035 (2)	0.0204 (6)	
H2A	1.2800	0.6048	0.6739	0.024*	
C3	1.2633 (3)	0.5524 (2)	0.5155 (2)	0.0202 (6)	
H3A	1.3536	0.4845	0.5254	0.024*	
C4	1.1749 (3)	0.5801 (2)	0.4112 (2)	0.0198 (6)	
C5	1.0430 (3)	0.6791 (2)	0.3917 (2)	0.0197 (5)	
H5A	0.9847	0.6977	0.3207	0.024*	
C6	0.9991 (3)	0.7504 (2)	0.4805 (2)	0.0192 (5)	
C7	0.8817 (3)	0.8802 (2)	0.5908 (2)	0.0189 (5)	
C8	0.7550 (3)	0.9748 (2)	0.6377 (2)	0.0191 (5)	
C9	0.6588 (3)	1.0655 (2)	0.5555 (2)	0.0215 (6)	
H9A	0.6823	1.0685	0.4716	0.026*	
C10	0.5302 (3)	1.1510 (3)	0.5944 (2)	0.0230 (6)	
H10A	0.4667	1.2129	0.5380	0.028*	
C11	0.4958 (3)	1.1445 (2)	0.7171 (2)	0.0217 (6)	
C12	0.5862 (3)	1.0553 (3)	0.8008 (2)	0.0246 (6)	
H12A	0.5601	1.0519	0.8846	0.029*	
C13	0.7156 (3)	0.9708 (3)	0.7609 (2)	0.0234 (6)	
H13A	0.7784	0.9093	0.8181	0.028*	
C14	1.0648 (3)	0.8116 (2)	0.7687 (2)	0.0192 (5)	
C15	1.1250 (3)	0.9200 (3)	0.7822 (2)	0.0231 (6)	
H15A	1.1384	0.9889	0.7151	0.028*	
C16	1.1652 (4)	0.9260 (3)	0.8952 (3)	0.0276 (6)	
H16A	1.2042	1.0003	0.9063	0.033*	
C17	1.1488 (3)	0.8242 (3)	0.9922 (2)	0.0273 (6)	
H17A	1.1755	0.8293	1.0695	0.033*	
C18	1.0937 (3)	0.7152 (3)	0.9766 (3)	0.0270 (6)	
H18A	1.0854	0.6449	1.0429	0.032*	
C19	1.0505 (3)	0.7082 (3)	0.8650 (2)	0.0216 (6)	
H19A	1.0116	0.6338	0.8542	0.026*	
C20	1.2139 (3)	0.5004 (2)	0.3189 (2)	0.0191 (5)	
C21	1.3934 (4)	0.3264 (3)	0.2602 (3)	0.0264 (6)	
H21A	1.2869	0.2967	0.2490	0.032*	
H21B	1.4691	0.2515	0.2994	0.032*	
C22	1.4756 (4)	0.3817 (3)	0.1393 (3)	0.0314 (7)	
H22A	1.5059	0.3153	0.0926	0.047*	
H22B	1.5781	0.4155	0.1496	0.047*	
H22C	1.3968	0.4504	0.0963	0.047*	
C23	0.3683 (3)	1.2831 (3)	0.8431 (2)	0.0243 (6)	

### Atomic displacement parameters (Å^2^)

**Table:**

	*U*^11^	*U*^22^	*U*^33^	*U*^12^	*U*^13^	*U*^23^
F1	0.0296 (9)	0.0283 (9)	0.0357 (10)	0.0098 (7)	0.0045 (7)	−0.0080 (7)
F2	0.0390 (10)	0.0321 (10)	0.0271 (9)	0.0045 (8)	0.0087 (7)	−0.0028 (7)
F3	0.0253 (9)	0.0341 (10)	0.0577 (12)	−0.0051 (7)	0.0036 (8)	−0.0194 (9)
O1	0.0208 (9)	0.0244 (10)	0.0220 (10)	0.0033 (7)	0.0008 (7)	−0.0077 (8)
O2	0.0235 (10)	0.0285 (11)	0.0222 (10)	−0.0004 (8)	−0.0027 (8)	−0.0071 (8)
O3	0.0225 (10)	0.0368 (12)	0.0281 (11)	0.0106 (8)	−0.0037 (8)	−0.0125 (9)
N1	0.0180 (10)	0.0213 (12)	0.0201 (11)	−0.0009 (9)	0.0020 (9)	−0.0022 (9)
N2	0.0175 (10)	0.0197 (11)	0.0190 (11)	0.0012 (8)	0.0008 (8)	−0.0035 (9)
C1	0.0163 (12)	0.0209 (13)	0.0185 (13)	−0.0044 (10)	0.0022 (10)	−0.0054 (10)
C2	0.0190 (12)	0.0238 (14)	0.0175 (13)	−0.0027 (10)	−0.0020 (10)	−0.0026 (10)
C3	0.0162 (12)	0.0216 (14)	0.0208 (13)	−0.0012 (10)	0.0025 (10)	−0.0033 (11)
C4	0.0191 (12)	0.0185 (13)	0.0204 (13)	−0.0054 (10)	0.0057 (10)	−0.0034 (10)
C5	0.0184 (12)	0.0231 (14)	0.0165 (13)	−0.0027 (10)	−0.0011 (10)	−0.0022 (10)
C6	0.0147 (11)	0.0202 (13)	0.0206 (13)	−0.0012 (10)	0.0032 (10)	−0.0034 (10)
C7	0.0165 (12)	0.0202 (13)	0.0183 (13)	−0.0034 (10)	−0.0013 (10)	−0.0003 (10)
C8	0.0172 (12)	0.0185 (13)	0.0213 (13)	−0.0033 (10)	−0.0003 (10)	−0.0042 (10)
C9	0.0214 (13)	0.0257 (14)	0.0159 (13)	−0.0037 (10)	0.0016 (10)	−0.0030 (11)
C10	0.0196 (13)	0.0245 (14)	0.0228 (14)	−0.0003 (10)	−0.0038 (10)	−0.0016 (11)
C11	0.0163 (12)	0.0238 (14)	0.0243 (14)	0.0020 (10)	0.0016 (10)	−0.0086 (11)
C12	0.0211 (13)	0.0319 (16)	0.0185 (13)	0.0013 (11)	0.0008 (10)	−0.0054 (11)
C13	0.0213 (13)	0.0242 (14)	0.0211 (14)	0.0010 (11)	−0.0018 (10)	−0.0004 (11)
C14	0.0143 (12)	0.0242 (14)	0.0172 (13)	0.0028 (10)	0.0005 (10)	−0.0050 (10)
C15	0.0196 (13)	0.0245 (14)	0.0235 (14)	−0.0021 (10)	0.0030 (10)	−0.0046 (11)
C16	0.0237 (14)	0.0324 (16)	0.0289 (15)	−0.0056 (12)	0.0002 (11)	−0.0112 (13)
C17	0.0203 (13)	0.0425 (18)	0.0198 (14)	0.0028 (12)	−0.0029 (11)	−0.0121 (12)
C18	0.0198 (13)	0.0331 (16)	0.0230 (14)	0.0011 (11)	0.0012 (11)	−0.0001 (12)
C19	0.0161 (12)	0.0214 (13)	0.0249 (14)	0.0021 (10)	0.0009 (10)	−0.0047 (11)
C20	0.0174 (12)	0.0199 (13)	0.0178 (13)	−0.0028 (10)	0.0034 (10)	−0.0015 (10)
C21	0.0267 (14)	0.0229 (15)	0.0302 (15)	0.0027 (11)	−0.0028 (12)	−0.0109 (12)
C22	0.0265 (15)	0.0397 (18)	0.0287 (16)	−0.0009 (13)	0.0022 (12)	−0.0132 (13)
C23	0.0212 (13)	0.0245 (14)	0.0249 (14)	0.0000 (10)	0.0001 (11)	−0.0035 (11)

### Geometric parameters (Å, º)

**Table:**

F1—C23	1.326 (3)	C9—C10	1.385 (4)
F2—C23	1.339 (3)	C9—H9A	0.9500
F3—C23	1.331 (3)	C10—C11	1.385 (4)
O1—C20	1.357 (3)	C10—H10A	0.9500
O1—C21	1.449 (3)	C11—C12	1.380 (4)
O2—C20	1.205 (3)	C12—C13	1.386 (4)
O3—C23	1.328 (3)	C12—H12A	0.9500
O3—C11	1.416 (3)	C13—H13A	0.9500
N1—C7	1.314 (3)	C14—C15	1.388 (4)
N1—C6	1.392 (3)	C14—C19	1.390 (4)
N2—C1	1.388 (3)	C15—C16	1.386 (4)
N2—C7	1.390 (3)	C15—H15A	0.9500
N2—C14	1.434 (3)	C16—C17	1.386 (4)
C1—C2	1.389 (4)	C16—H16A	0.9500
C1—C6	1.403 (4)	C17—C18	1.383 (4)
C2—C3	1.381 (4)	C17—H17A	0.9500
C2—H2A	0.9500	C18—C19	1.383 (4)
C3—C4	1.408 (4)	C18—H18A	0.9500
C3—H3A	0.9500	C19—H19A	0.9500
C4—C5	1.389 (4)	C21—C22	1.494 (4)
C4—C20	1.491 (3)	C21—H21A	0.9900
C5—C6	1.398 (3)	C21—H21B	0.9900
C5—H5A	0.9500	C22—H22A	0.9800
C7—C8	1.479 (3)	C22—H22B	0.9800
C8—C13	1.400 (4)	C22—H22C	0.9800
C8—C9	1.402 (4)		
			
C20—O1—C21	115.6 (2)	C12—C13—C8	121.0 (2)
C23—O3—C11	118.9 (2)	C12—C13—H13A	119.5
C7—N1—C6	105.1 (2)	C8—C13—H13A	119.5
C1—N2—C7	105.7 (2)	C15—C14—C19	121.2 (2)
C1—N2—C14	124.9 (2)	C15—C14—N2	120.0 (2)
C7—N2—C14	129.3 (2)	C19—C14—N2	118.8 (2)
N2—C1—C2	131.5 (2)	C16—C15—C14	118.8 (3)
N2—C1—C6	105.9 (2)	C16—C15—H15A	120.6
C2—C1—C6	122.6 (2)	C14—C15—H15A	120.6
C3—C2—C1	117.1 (2)	C17—C16—C15	120.4 (3)
C3—C2—H2A	121.4	C17—C16—H16A	119.8
C1—C2—H2A	121.4	C15—C16—H16A	119.8
C2—C3—C4	121.0 (2)	C18—C17—C16	120.2 (3)
C2—C3—H3A	119.5	C18—C17—H17A	119.9
C4—C3—H3A	119.5	C16—C17—H17A	119.9
C5—C4—C3	121.8 (2)	C19—C18—C17	120.2 (3)
C5—C4—C20	116.9 (2)	C19—C18—H18A	119.9
C3—C4—C20	121.3 (2)	C17—C18—H18A	119.9
C4—C5—C6	117.5 (2)	C18—C19—C14	119.1 (3)
C4—C5—H5A	121.2	C18—C19—H19A	120.4
C6—C5—H5A	121.2	C14—C19—H19A	120.4
N1—C6—C5	130.0 (2)	O2—C20—O1	122.9 (2)
N1—C6—C1	110.0 (2)	O2—C20—C4	124.9 (2)
C5—C6—C1	120.0 (2)	O1—C20—C4	112.2 (2)
N1—C7—N2	113.3 (2)	O1—C21—C22	113.5 (2)
N1—C7—C8	121.9 (2)	O1—C21—H21A	108.9
N2—C7—C8	124.5 (2)	C22—C21—H21A	108.9
C13—C8—C9	118.2 (2)	O1—C21—H21B	108.9
C13—C8—C7	123.1 (2)	C22—C21—H21B	108.9
C9—C8—C7	118.4 (2)	H21A—C21—H21B	107.7
C10—C9—C8	121.2 (2)	C21—C22—H22A	109.5
C10—C9—H9A	119.4	C21—C22—H22B	109.5
C8—C9—H9A	119.4	H22A—C22—H22B	109.5
C11—C10—C9	118.8 (2)	C21—C22—H22C	109.5
C11—C10—H10A	120.6	H22A—C22—H22C	109.5
C9—C10—H10A	120.6	H22B—C22—H22C	109.5
C12—C11—C10	121.7 (2)	F1—C23—O3	108.2 (2)
C12—C11—O3	123.0 (2)	F1—C23—F3	108.7 (2)
C10—C11—O3	115.2 (2)	O3—C23—F3	113.3 (2)
C11—C12—C13	119.1 (2)	F1—C23—F2	107.1 (2)
C11—C12—H12A	120.4	O3—C23—F2	113.0 (2)
C13—C12—H12A	120.4	F3—C23—F2	106.4 (2)
			
C7—N2—C1—C2	−178.7 (3)	C9—C10—C11—C12	−0.1 (4)
C14—N2—C1—C2	1.3 (4)	C9—C10—C11—O3	−177.8 (2)
C7—N2—C1—C6	1.3 (3)	C23—O3—C11—C12	38.5 (4)
C14—N2—C1—C6	−178.7 (2)	C23—O3—C11—C10	−143.8 (3)
N2—C1—C2—C3	178.9 (2)	C10—C11—C12—C13	0.5 (4)
C6—C1—C2—C3	−1.1 (4)	O3—C11—C12—C13	178.1 (2)
C1—C2—C3—C4	0.3 (4)	C11—C12—C13—C8	−0.1 (4)
C2—C3—C4—C5	0.6 (4)	C9—C8—C13—C12	−0.8 (4)
C2—C3—C4—C20	−176.7 (2)	C7—C8—C13—C12	−174.7 (2)
C3—C4—C5—C6	−0.7 (4)	C1—N2—C14—C15	117.3 (3)
C20—C4—C5—C6	176.8 (2)	C7—N2—C14—C15	−62.7 (4)
C7—N1—C6—C5	177.8 (3)	C1—N2—C14—C19	−64.2 (3)
C7—N1—C6—C1	0.2 (3)	C7—N2—C14—C19	115.8 (3)
C4—C5—C6—N1	−177.7 (3)	C19—C14—C15—C16	−2.4 (4)
C4—C5—C6—C1	−0.2 (4)	N2—C14—C15—C16	176.1 (2)
N2—C1—C6—N1	−1.0 (3)	C14—C15—C16—C17	1.3 (4)
C2—C1—C6—N1	179.1 (2)	C15—C16—C17—C18	0.6 (4)
N2—C1—C6—C5	−178.9 (2)	C16—C17—C18—C19	−1.6 (4)
C2—C1—C6—C5	1.1 (4)	C17—C18—C19—C14	0.6 (4)
C6—N1—C7—N2	0.8 (3)	C15—C14—C19—C18	1.4 (4)
C6—N1—C7—C8	−173.6 (2)	N2—C14—C19—C18	−177.1 (2)
C1—N2—C7—N1	−1.4 (3)	C21—O1—C20—O2	−4.3 (4)
C14—N2—C7—N1	178.6 (2)	C21—O1—C20—C4	174.5 (2)
C1—N2—C7—C8	172.8 (2)	C5—C4—C20—O2	−7.8 (4)
C14—N2—C7—C8	−7.2 (4)	C3—C4—C20—O2	169.6 (3)
N1—C7—C8—C13	151.2 (3)	C5—C4—C20—O1	173.4 (2)
N2—C7—C8—C13	−22.5 (4)	C3—C4—C20—O1	−9.2 (3)
N1—C7—C8—C9	−22.7 (4)	C20—O1—C21—C22	79.5 (3)
N2—C7—C8—C9	163.6 (2)	C11—O3—C23—F1	166.4 (2)
C13—C8—C9—C10	1.3 (4)	C11—O3—C23—F3	45.8 (3)
C7—C8—C9—C10	175.5 (2)	C11—O3—C23—F2	−75.2 (3)
C8—C9—C10—C11	−0.9 (4)		

### Hydrogen-bond geometry (Å, º)

*Cg*1 and *Cg*2 are the centroids of the C14–C19 and N1/N2/C1/C6/C7 rings, respectively.

**Table:**

*D*—H···*A*	*D*—H	H···*A*	*D*···*A*	*D*—H···*A*
C12—H12*A*···F2	0.95	2.37	2.956 (3)	120
C15—H15*A*···N1^i^	0.95	2.56	3.490 (3)	167
C18—H18*A*···O2^ii^	0.95	2.40	3.307 (4)	160
C19—H19*A*···O2^iii^	0.95	2.50	3.412 (3)	160
C13—H13*A*···*Cg*1	0.95	2.79	3.592 (3)	142
C21—H21*A*···*Cg*2^iii^	0.99	2.95	3.634 (3)	127

Symmetry codes: (i) −*x*+2, −*y*+2, −*z*+1; (ii) *x*, *y*, *z*+1; (iii) −*x*+2, −*y*+1, −*z*+1.
